# Association of vulvar lichen sclerosus with endometrial and ovarian cancer

**DOI:** 10.1016/j.jdin.2022.07.003

**Published:** 2022-07-22

**Authors:** Anand K. Ganesan, Thomas H. Taylor, Christina N. Kraus

**Affiliations:** a Department of Dermatology, University of California, Irvine, Irvine, California; b Department of Epidemiology and Biostatistics, University of California, Irvine, Irvine, California; c Department of Biostatistics, University of California, Irvine, Chao Family Comprehensive Cancer Center, Orange, California

**Keywords:** endometrial cancer, gynecologic dermatology, lichen sclerosus, ovarian cancer, vulvar

*To the Editor:* Lichen sclerosus (LS) is a chronic inflammatory dermatosis of largely unknown pathogenesis with a predilection for vulvar skin. LS is associated with vulvar malignancy and a recent report links LS and breast cancer (BC),^1^ perhaps through epitope spread. Here, we assessed associations between LS and 4 following types of cancer: endometrial carcinoma (EC), ovarian carcinoma (OC), BC, and colorectal cancer (CC). Endometrial stromal cells and ovarian follicles express extracellular matrix protein 1,^2^^,^^3^ a suspected etiologic agent in LS. We included CC as a negative control and BC to try to replicate the recent finding.

A retrospective, survey study of all patients seen at our institution between 2011 and 2021 identified 369 women aged at least 54 years with LS. The control group had postmenopausal atrophic vaginitis (genitourinary syndrome of menopause, [GSM]), which has no known predisposition to malignancies, and is, commonly diagnosed by gynecologists. Diagnoses were by The International Classification of Diseases, 10th Revision codes (LS:L90; GSM:N95.2; EC:C54.1,C55; OC:C56; BC:C50; CC:C18-20). We disregarded the temporal order of LS and cancer diagnoses. Individual charts were reviewed to confirm the accuracy of LS and malignancy diagnoses, age, LS location, history of other malignancies, treatment, and medications. Subsequently, we repeated our analyses on data from the University of California-wide deidentified data set, in which we could not confirm the diagnoses. The strength of the association was estimated using odds ratios and 95% confidence intervals.

At our institution, LS was significantly associated with EC and OC, but not BC or CC, compared with GSM (Table I). Of 29 patients with LS and EC, 28 had vulvar LS and 1 had extragenital LS. All patients with OC had vulvar LS. Eleven patients underwent radiation for EC and 2 patients for OC. The University of California-wide data confirmed an association between LS and both EC and OC, but not BC (Table I).

**Table I:** Associations between LS and selected cancer types in the smaller, institutional data set (upper panel) and the larger, UC-wide, data set (lower panel)

Institutional study population				
Malignancy	Patients with LS (n = 369) (%)	Patients with GSM (n = 3995) (%)	Likelihood ratio, χ^2^	OR (95% CI)
Endometrial	29 (7.9)	177 (4.4)	7.55, *P* < .006	1.84 (1.22-2.77)
Ovarian	12 (3.3)	53 (1.3)	6.60, *P* < .01	2.50 (1.32-4.73)
Breast	28 (7.6)	286 (7.2)	0.09, *P* < .77	1.06 (0.71-1.60)
Colorectal	3 (0.8)	44 (1.1)	0.29, *P* < .60	0.74 (0.22-2.39)

The University of California-Wide deidentified study population				
Malignancy	Patients with LS (n = 2057) (%)	Patients with GSM (n = 53,036) (%)	Likelihood ratio, χ^2^	OR (95% CI)
Endometrial	40 (1.9)	618 (1.2)	8.63, *P* < .0033	1.68 (1.21-2.33)
Ovarian	34 (1.7)	589 (1.1)	4.59, *P* < .0322	1.50 (1.05-2.13)
Breast	181 (8.8)	4949 (9.3)	0.66, *P* < .417	0.94 (0.80-1.10)
Colorectal	17 (0.8)	513 (1.0)	0.43, *P* < .511	0.85 (0.52-1.39)

*CI*, Confidence interval; *GSM*, genitourinary syndrome of menopause; *LS*, lichen sclerosus; *OR*, odds ratio.

When gynecologic cancer precedes LS, cancer therapies may increase the risk of LS through increasing levels of circulating extracellular matrix protein 1, and radiotherapy effects and estrogen status. When LS precedes cancer, mechanisms of connection are more elusive. Our null finding with BC may arise from differences in study power or patient population.

These data suggest that LS is associated with EC and OC, in addition to vulvar cancers. We did not replicate a report linking LS with BC and found no association between LS and CC. This pattern of results suggests that LS may be linked to gynecologic cancers, as distinguished from other cancer types. We cannot infer the cause from this study: perhaps, LS and certain cancers have similar underlying susceptibilities, or one arises from treatment or observation of the other. The limitations of the study include the risk of confounding inherent in the data, delay in diagnosis of LS, and that our groups may have had differential access to care or evaluation for LS before their diagnosis of malignancy. Other limitations include the retrospective, survey nature of the study, limited information on potentially important covariates, and the small number of cases. Prospective studies with complete and accurate information on diagnosis, temporal order, and plausibly important covariates are necessary to improve our understanding of the relationship between LS and gynecologic malignancies.

## Conflicts of interest

None disclosed.
